# Treatment of gastrointestinal stromal tumours in paediatric and young adult patients with sunitinib: a multicentre case series

**DOI:** 10.1186/s12885-017-3727-1

**Published:** 2017-11-06

**Authors:** Piotr Rutkowski, Heather Magnan, Alexander J. Chou, Charlotte Benson

**Affiliations:** 1 Maria Skłodowska-Curie Institute – Oncology Center, Roentgena 5, 02-781 Warsaw, Poland; 2 Memorial Sloan Kettering Cancer Center, New York, NY USA; 3 The Royal Marsden Hospital, London, UK

**Keywords:** Paediatric, Young adult, GIST, Case series, Sunitinib

## Abstract

**Background:**

Gastrointestinal stromal tumours (GIST) are rarely encountered mesenchymal tumours of the gastrointestinal tract (1.5 people per 100,000/year) that are even more rarely seen in paediatric patients (1–2% of all cases). The standard treatment for advanced adult GIST is imatinib with sunitinib as a second-line option. Although the efficacy and tolerability of sunitinib in adults with GIST has been established, little is known of the profile of sunitinib in paediatric/young adult patients with GIST given the rarity of this disease.

**Methods:**

Paediatric/young adult patients aged up to 21 years with diagnosis of GIST who were treated with sunitinib were identified from retrospective records from three centres in Europe and the US. Most patients commenced sunitinib in a 6-week cycle, however, dosing could be reduced, delayed, changed to (or initiated with) a continuous schedule. Objective response (Response Evaluation Criteria In Solid Tumours [RECIST]) and adverse events were recorded.

**Results:**

We identified 9 paediatric/young adult patients (aged 11–21 years) with GIST who were treated with sunitinib de novo (*n* = 1) or following failure of imatinib (*n* = 8). Progressive disease was previously documented for all patients including 7 patients during imatinib therapy. Baseline patient and tumour profile characteristics showed a distinct profile (notably all were wild-type *KIT/PDGFR*) compared to that established for adults. Sunitinib treatment was associated with a best response of stable disease for 7 patients, with disease stabilisation lasting from 1 month to >73 months and a median progression free survival time of 15 months. There was some evidence of better disease control for sunitinib when compared to prior imatinib. Most adverse events with sunitinib were manageable and all were consistent with the known profile of the agent.

**Conclusion:**

The ability to draw firm conclusions from this case series is limited by the small number of patients and the use of retrospective data which is largely reflective of the rarity of this condition. However, our findings provide initial evidence of clinical benefit and a generally manageable toxicity profile for sunitinib when administered to paediatric/young adult patients with GIST, most of whom had documented progressive disease during prior imatinib treatment.

## Background

Gastrointestinal stromal tumours (GIST) are mesenchymal tumours that arise in the gastrointestinal tract, most often in the stomach (60% of patients) or the jejunum/ileum (30%), and less so in the duodenum, colon, rectum, appendix and oesophagus (<1–5%) [1]. These are rare tumours, with an estimated incidence of 1.5 per 100,000 people every year [2] that more often occur in the ageing population (median age at diagnosis 60–65 years) [3]. Pathological diagnosis of GIST relies on morphology and immunohistochemistry (CD117 and/or DOG1), although analysis for *KIT* and *PDGFRA* mutations are useful for confirming diagnosis [4]. Prognostic factors are the mitotic rate, tumour size and site (gastric GISTs have a better prognosis than small bowel or rectal GISTs [1]). Furthermore, mutational analysis also has a prognostic value and can predict sensitivity to targeted therapy [4].

Localised disease is usually treated by surgical excision, with surgical margins and tumour rupture additional prognostic factors [4]. Patients considered at high risk of recurrence should receive adjuvant therapy with imatinib 400 mg/day for three years [4], and the same imatinib dose is used for patients with locally advanced inoperable or metastatic disease [5–8]. Patients with a *KIT* exon 9 mutation have fared better on imatinib 800 mg/day [9] and escalation to this higher dose is also an option in the event of relapse [5–7]. Treatment should be continued indefinitely, as cessation can lead to rapid tumour progression [10].

In patients who progress or who are rarely intolerant to imatinib, second line therapy with sunitinib 50 mg/day every 4 weeks with a rest period of two weeks is standard practice [11, 12] with 37.5 mg/day continuous therapy an alternative [13]. This latter approach has been adopted following regulatory approval of the intermittent regimen. In the case of failure during treatment with sunitinib, regorafenib 160 mg/day every 3 out of 4 weeks has shown clinical benefit [14]. Beyond third-line therapy, participation in clinical trials is encouraged [4], although rechallenge with imatinib has also shown benefit [15]. Furthermore, continuation of treatment with a tyrosine kinase inhibitor may slow disease progression after documented progressive disease [4].

Paediatric patients with GIST represent an extremely rare and characteristic sub-population. Paediatric GIST has been noted to account for about only 1 to 2% of all GIST cases [16, 17]. These patients tend to be predominantly female, with gastric disease, possible lymph node metastasis and the absence of either *KIT* or *PDGRFA* mutations [3]. However, germline autosomal dominant mutations of KIT have been associated with familial multiple GISTs in paediatric patients [4]. Deficiency in one or more subunits of succinate dehydrogenase (SDH) has also been linked with GIST in Carney Triad syndrome (marked by gastric GIST, paraganglioma and pulmonary chondromas) [18] and in Carney-Stratakis syndrome (characterised by GIST and paraganglioma) [19, 20]. Furthermore, recent work has identified three distinct wild-type GIST subtypes based on SDH which may have implications for prognosis and treatment [21]. Against this background, the varied GIST genotypes associated with paediatric GIST present notable diagnostic and therapeutic challenges [22].

Although the efficacy and tolerability of sunitinib in adult patients with GIST has been established [11, 13], little is known of the profile of sunitinib in paediatric patients with GIST, with published reports limited to either case studies or a small cohort of cases [22, 23]. Here we describe a series of paediatric/young adult patients with GIST treated with sunitinib, most following imatinib failure.

## Methods

Paediatric/young adult patients aged up to 21 years with diagnosis of GIST were identified from retrospective records from three centres; Maria Skłodowska-Curie Institute – Oncology Center, Warsaw, Poland; Memorial Sloan Kettering, New York, USA; and The Royal Marsden Hospital, London, UK. Data were compiled and reported broadly in accordance with the “Care Guidelines” on individual case reports [24]. At diagnosis, age and gender were recorded. Baseline data (start of sunitinib treatment) compiled included age, gender, height, bodyweight, site of primary and any metastatic tumours, tumour morphology, tumour size, whether or not the tumour was multifocal, prior treatment, *KIT/PDGFRA* mutation status and other clinical features of relevance. Of note, genomic screening was performed for the presence of KIT (exons 9, 11, 13 and 17) and *PDGFRA* (exons 12, 14, and 18) gene mutations based on DNA isolated from paraffin-embedded or fresh frozen tumour tissue [25].

The majority of patients were treated with sunitinib in a 6-week cycle from the start, however, dosing could be reduced, delayed or changed to (or initiated with) a continuous schedule to optimize the benefit-risk profile according to decision of the treating physician. Of note, dose escalation beyond that used in the standard regimen was not an option in the UK. Treatment was continued until confirmed progression of the disease or unacceptable toxicity. Routine physical, clinical and laboratory test assessments were performed at follow-up every 4–8 weeks. The objective response of GIST to sunitinib therapy was evaluated with serial CT examinations (performed every 2–3 months), according to Response Evaluation Criteria in Solid Tumors (RECIST). If progression was noted, patients were treated with other tyrosine kinase inhibitors or cytotoxic chemotherapy, or best supportive care only. Due consideration was also given to inclusion in a clinical trial for treatment with an experimental agent.

## Results

A total of 9 paediatric/young adult patients with a diagnosis of GIST between 2001 and 2013 who were treated with sunitinib were identified from clinical records at the Maria Skłodowska-Curie Memorial Cancer Center and Institute of Oncology, Warsaw (*n* = 4), Memorial Sloan Kettering, New York (*n* = 3), and The Royal Marsden Hospital, London (*n* = 2). Of these 9 patients, the majority were female (*n* = 6) and age at diagnosis ranged from 11 to 21 years, although all but 1 patient was aged ≤18 years: age at start of sunitinib treatment ranged from 12 to 21 years with all but 3 patients aged ≤18 years. Patient height ranged from 148 cm to 176 cm, bodyweight from 39 kg to 62 kg and body surface area from 1.28 m^2^ 1.68 m^2^. All patients had a primary gastric tumour, and all but 2 had metastatic disease in the liver and/or abdomen (Table 1). Presentation involved overt or occult gastrointestinal bleeding in 7, while a further patient had evidence of marked anaemia. Other presentation signs and symptoms included pain, nausea and vomiting in 1 individual. All patients had documented progressive disease. Pathological examination of tumour samples revealed that 4 patients had tissue containing epithelioid cells, 2 patients had tissue with spindle cells and the remaining 3 a mixed morphology. There were no mutations in either *KIT* or *PDGFR* for any patient.

**Table 1:** Patient characteristics^a^

	Site of primary (P) and metastases (M)	Prior treatment	Morphology	Multifocal (MF) Tumour size Mitotic index (MI)	KIT/PDGFRA mutation status	Other clinical features of particular relevance
Patient A	P – Stomach M – Liver	R2 surgery, imatinib	Epithelioid	MF- Yes 11.0 cm MI >10/50 HPF	Both WT	GI bleeding, palpable tumour
Patient B	P – Stomach M– Liver – IP cavity	R2 surgery then imatinib after recurrence	Mixed	MF –no 3.0 cm MI 6/50 HPF	Both WT	GI bleeding
Patient C	P – Stomach M – Liver – IP Cavity	R2 surgery, imatinib	Epithellioid	MF – yes 6.0 cm MI NA	Both WT	GI bleeding
Patient D	P – Stomach – IP cavity	R2 surgery, imatinib	Mixed	MF – yes 11.0 cm MI 05/50 HPF	Both WT	GI bleeding
Patient E	P – Stomach M – Omentum	Surgery, imatinib at first progression	Mixed	MF – Yes 7.0 cm 10/50 HPF	Both WT	GI bleeding from tumour
Patient F	P – Stomach M – None	Surgery at diagnosis and recurrence	Epithelioid	MF – No 4.5 cm MI 50//10 HPF	Both WT	Pain, nausea, vomiting, GI bleeding from tumour
Patient G	P – Stomach M – None	Surgery	Mixed	MF – No 10.0 cm MI 17/50 HPF	Both WT	Abdominal pain
Patient H	P – Stomach M – Liver – IP cavity	Imatinib	Spindle	MF-yes NA MI 1/20 HP	Both WT	Marked anaemia
Patient I	P – Stomach M – Liver	Surgery, imatinib	Spindle	MF-no 3 cm MI 20/50 HPF	Both WT	Melaena and anaemia

^a^To maintain patient anonymity individual data relating to age, gender, height, bodyweight, date of diagnosis, age at diagnosis and start of sunitinib therapy are summarised in the results section

HPF = high powered field; GI = gastrointestinal; IP = intraperitoneal; NA = not available; R2 = macroscopic residual tumour on resection; WT = wild type

Before commencing sunitinib, all but 1 patient had been treated with surgery and all but one patient had received imatinib (Table 1). Of the 8 patients that received imatinib at a dosage of 400 mg/day, 3 patients required dose reductions and 1 patient received dose escalation to 800 mg/day (Table 2). Of note, dose escalation was not an option in the UK. All but one patient experienced stable disease as the best response and all but one patient discontinued imatinib due to documented progressive disease. The time to progression (TTP)/progression free survival (PFS) duration in this latter group ranged from 2 to 17 months (Table 2). The remaining patient who discontinued due to imatinib intolerance had a PFS duration of 7 months.

**Table 2:** Treatment and outcomes

	Imatinib response/dose/treatment duration	Imatinib PFS and TTP	Sunitinib response/dose/treatment duration/BSA	Sunitinib PFS and TTP	Overall follow up	Outcome	Adverse event/Toxicity during sunitinib treatment
Patient A	SD then PD; 400 mg OD then 300 mg OD; 8mo	PFS 17mo TTP 17mo	SD liver metastasis; 50 mg OD 4/2 schedule, then 37.5 mg OD; 73mo; 1.52m^2^	PFS 73mo TTP No progression	163mo	AWD on sunitinib since 2008	Hand and foot syndrome /TG 1 Arterial hypertension/TG 1 Skin rash/TG 2 Anaemia/TG 2 Diarrhoea/TG 2 Hypothyroidism/TG 2
Patient B	SD then PD; 400 mg OD; 9mo	PFS 8mo TTP 8mo	SD then PD; 37.5 mg OD then 50 mg OD 4/2 schedule; 7mo; 1.39m^2^	PFS 6mo TTP 6mo	159mo	AWD on regorafenib	Hypothyroidism/ TG 1 Diarrhoea/ TG 2 Skin rash/ TG 1
Patient C	SD then PD; 400 mg OD then 200 mg OD; 9mo	PFS 8mo TTP 8mo	SD then PD; 25 mg OD then 37.5 mg OD then 25 mg OD; 23 months; 1.28m^2^	PFS 6mo TTP 6mo	88mo	AWD on imatinib/doxorubicin	Cholecystitis /TG 3 Diarrhoea/TG 1
Patient D	PD; 400 mg OD; 12mo	PFS 12mo TTP 12mo	SD then PD; 50 mg OD 4/2 schedule then 37.5 mg OD; 24mo; 1.39m^2^	PFS 23mo TTP 23mo	260mo	AWD on treatment (nilotinib, then imatinib + doxorubicin, then imatinib 800 mg OD	Hypothyroidism/TG 3 Pyrosis/TG 1 Anaemia/TG 3 Thrombocytopenia/TG 2 Fatigue/TG 2 Amenorrhoea/TG 2 Discoloration of skin and hair/TG 2 Hand and foot syndrome/TG 2
Patient E	SD; 400 mg BID then 200 mg OD then 300 mg OD; 7mo	PFS 7mo TTP NA – intolerance (neutropenia, fatigue, joint pain)	PD; 25 mg OD 4/2 schedule (reduced due to prior intolerance of imatinib); 5mo; 1.51m^2^	PFS 5mo TTP 5mo	139mo	AWD, PD on nilotinib after sunitinib. Slow PD- asymptomatic, no treatment since 2011	Abdominal pain/TG 2 Bone pain/TG 2 Fatigue/TG 2 Patient elected to discontinue sunitinib due to above side effects, PD then documented
Patient F	NA	NA	PD; 50 mg OD 4/2 schedule; 1mo; 1.50m^2^	PFS 1mo TTP 1mo	25mo	DOD, after sunitinib also failed trametinib, regorafenib, a phase 1 clinical trial and pazopanib	Fatigue/TG 1 Thrombocytopenia/TG 1
Patient G	SD then PD; 400 mg OD; 14mo	PFS 14mo TTP 14mo	SD then PD; 37.5 mg OD then discontinued for 3mo for toxicity then restarted 12.5 mg OD increasing to 25 mg OD; 17mo; 1.65m^2^	PFS 17mo TTP 17mo	76mo	AWD, no treatment since sunitinib and subsequent SD under observation from 2012	Fatigue/TG 1 Oedema/TG 2 Epistaxis/TG 1 Headaches/TG 1
Patient H	SD then PD; 400 mg OD then 800 mg OD: 49mo	PFS 9mo TTP 9mo	SD for duration but initial minor response; Initial dose 50 mg OD, lowest dose 12.5 mg OD; 17mo; 1.60m^2^	PFS 5mo TTP 5mo	173mo	AWD on imatinib	Fatigue/TG 3 Mucositis/TG 3 Diarrhoea/TG 3
Patient I	SD then PD; 400 mg OD; 1mo	PFS 2mo TTP 2mo	SD then PD then SD^a^; 50 mg OD then 37.5 mg OD; >42mo (RFA during treatment for liver metastases); ^b^1.68m^2^	PFS 28mo TTP 28mo	86mo	AWD on sunitinib	Mucositis /TG 2 Diarrhoea/TG 2 Hypertension/TG 2

^a^PD liver lesion treated with RFA; ^b^Last recorded July 2012

AWD = alive with disease; BSA = Body surface area at start of sunitinib treatment (a, last record; b, during therapy); DOD = dead of disease; OD = once daily; mo = months; NA = not applicable; PD = progressive disease; PFS = progression-free survival; RFA = Radiofrequency Ablation; SD = stable disease; TG = toxicity grade; TTP = time to progression; 4/2 schedule = 4 weeks of daily treatment and 2 weeks no treatment

Sunitinib dosage regimens varied reflecting the evolution from the 4-weeks on and 2-weeks off approach using a standard approved dosage of 50 mg/day to the alternative continuous regimen using 37.5 mg/day. In both cases, variations to the usual starting doses were used. Dosage reductions were instigated for 2 patients and 1 of these patients withdrew from therapy for 3 months due to toxicity. A best response of stable disease was experienced for 7 of the 9 patients during therapy with sunitinib and all but 1 patient eventually experienced progressive disease (Table 2). Among those who progressed, PFS and TTP duration ranged from 1 to 23 months, while 1 patient remained progression free after 73 months. Overall, median PFS was 15 months.

Most adverse events experienced by patients treated with sunitinib were manageable with appropriate clinical intervention including dosage amendments (Table 2). The majority of adverse events were grade 1 or 2, while grade 3 toxicities were recorded for 3 patients and included cholecystitis (*n* = 1), hypothyroidism and anaemia (*n* = 1) and fatigue, mucositis and diarrhoea (*n* = 1). One patient discontinued due to grade 2 abdominal/bone pain and fatigue, while another discontinued for 3 months due to grade 2 oedema and grade 1 fatigue, epistaxis and headache before resuming therapy.

Follow up in our study ranged from 25 months to 260 months. At the time of data cut-off (20th February 2016), 8 patients remain alive with disease, 2 continue to be treated with sunitinib (both with stable disease), 4 are being treated with targeted agents either alone or in combination with chemotherapy, and 2 patients have stopped treatment and continue to be monitored (Table 2). One patient died of disease during the course of therapy.

## Discussion

This retrospective analysis revealed 9 paediatric/young adult patients treated with sunitinib. Baseline characteristics were broadly in line with that expected for a population of GIST paediatric patients, with female predominance, gastric tumour location and no evidence of KIT or PDGFR mutations [3]. Indeed, the presence of the KIT/PDGFR wild type has been shown to dominate in paediatric GISTs, particularly among females, although some males have been shown to have KIT mutations and downstream KIT activation [26]. SDH-deficiency was not evaluated in our series given that diagnoses were made a considerable time before reporting and in most cases ≥7 years prior. Most of our patients had either an epithelioid or mixed morphology consistent with profiles reported in the literature [16, 17], although 2 patients (1 male aged 21 yrs. and 1 female aged 15 yrs) had spindle morphology. Metastatic spread to locoregional lymph nodes is also a common feature of paediatric GIST [17] which was consistent with the findings in our series as well.

In this case series, sunitinib dosage regimens varied reflecting the evolution from the 4-weeks on and 2-weeks off approach using a standard approved dosage of 50 mg/day to the alternative continuous regimen using 37.5 mg/day, with further variations to the usual starting doses used. Of note, an ongoing multicentre global study of sunitinib in paediatric and young adults with GIST is using a starting dose of 15 mg/m^2^ for paediatric patients aged 6–18 years with the option of dose escalation, and 50 mg every day for young adults aged 18–21 years, both schedules using the 4 weeks on and 2 weeks off schedule (NCT01396148). Physicians treating patients in our case series did not specifically select a sunitinib dose based on bodyweight or body surface area, although these aspects were considered when deciding on dose selection. Retrospective analysis of dose by body surface area at baseline (where available) and outcome (efficacy or toxicity) showed no obvious correlation in this small sample.

A best response of stable disease was experienced for 7 of 9 patients during therapy with sunitinib although all but 1 patient eventually experienced progressive disease. Overall, median PFS was 15 months which appears to be favourable when compared with a median PFS of 27–34 weeks in adults [11, 13], although this apparent difference may reflect the indolent nature of the disease [26]. Three patients showed more than a doubling of PFS/TTP with sunitinib compared with imatinib, but where imatinib showed an advantage on these measures the differences were less stark. These findings are broadly in line with those of a study which evaluated 7 paediatric patients (age range 10–17 yrs) with imatinib-resistant GIST who were treated with the intermittent sunitinib regimen [23]. A best response of partial response was recorded for one patient, 5 patients had stable disease and 1 patient progressed. Of note, disease stabilisation ranged from 7 to 21+ months (median 15 months) and TTP was longer on sunitinib than prior imatinib in 5 of the 6 cases. The pharmacological activity of sunitinib as a multi-kinase inhibitor targeting several tyrosine kinases including those of vascular endothelial growth factor receptor (*VEGFR)*, *PDGFR* and *KIT* [27], and clinical activity in GIST patients with wild-type *KIT* [28] is consistent with the activity of this agent in our case series.

It is worthy of note that all but 1 of our patients underwent surgery where the primary tumour was located in the stomach. Gastrectomy has been shown to have no influence on exposure to sunitinib, in contrast to findings for imatinib [29]. Whether or not this had any bearing on the findings of our study is open to question.

There has been some question around the contribution of *KIT* and *PDGFR* mutation status to the general clinical effectiveness of sunitinib in GIST, although the effectiveness of sunitinib as a post-imatinib therapy in GIST has been confirmed in a real-world use study regardless of mutation status [30]. All of our patients had wild-type GIST and 7 of the 9 patients had a best response of stable disease with PFS ranging from 1 to >73 months.

Most adverse events experienced by patients treated with sunitinib were manageable and the majority of adverse events were grade 1 or 2, while grade 3 toxicities were recorded for 3 patients; 2 patients discontinued due to grade 2 adverse events. These findings are consistent with the well defined toxicity profile for sunitinib in patients with GIST and the finding that side effects can be managed with standard medical intervention and/or dose modification in this population [31]. The tolerability and safety in a small series of paediatric GIST patients [23] was not dissimilar to that observed in our series.

Follow up in our study ranged from 25 months to 260 months and at data cut-off 8 patients remain alive with disease and 2 continue to be treated with sunitinib (both with stable disease). Paediatric GISTs, unlike those in adults, tend to follow a rather indolent course, despite a high rate of metastatic spread to the peritoneal cavity and liver [26]. Overall, the characteristics of our patients and our treatment experience support this observation.

## Conclusions

Our series of 9 patients confirms a distinctly different profile for paediatric/young adult GIST compared with adult GIST. Given the unpredictable natural course of paediatric GIST a first strategy is often to monitor patients in a scenario of “watchful waiting”. However, progressive disease was recorded for all of our patients before sunitinib therapy and also for 7 of the patients during imatinib therapy (with one patient discontinuing imatinib due to intolerance). Treatment with sunitinib in this population was associated with stable disease as a best response for 7 patients, with disease stabilisation lasting from 1 month to >73 months. Median PFS was 15 months which compares favourably to that recorded for adults although this difference may reflect the indolent nature of GIST in our patient population. Furthermore, some evidence of better disease control duration arose for sunitinib when compared to prior imatinib. Most adverse events experienced by patients treated with sunitinib were manageable with appropriate clinical intervention including dosage amendments and all were consistent with the established profile of the agent.

The ability to draw firm conclusions from this case series is limited by the small number of patients and the use of retrospective data outside of a formal clinical trial. Nevertheless, this also reflects the uniquely rare population of paediatric/young adult patients with GIST which in turn represent a challenge to clinical trial recruitment. A multicentre global clinical trial of sunitinib in paediatric and young adult patients with GIST is currently ongoing (NCT01396148). Until this and any further studies are completed, evidence from case series such as ours provides useful information where a definitive approach based on robust evidence-based recommendations are unlikely to emerge in the short term. Despite these shortcomings, our findings provide initial evidence of clinical benefit and a generally manageable toxicity profile for sunitinib when administered to paediatric and young adult patients with GIST, most of whom had documented progressive disease during prior treatment with imatinib.

## Abbreviations

AWD: Alive with disease
BSA: Body surface area
DOD: Dead of disease
DOG1: Discovery On GIST1
GI: Gastrointestinal
GIST: Gastrointestinal stromal tumours
HPF: High powered field
IP: Intraperitoneal
KIT: Proto-oncogene KIT (also known as CD117)
M: Metastases
MF: Multi-focal (disease)
Mo: Months
NA: Not available
OD: Once daily
P: Primary (tumour)
PD: Progressive disease
PDGFRA: Platelet-derived growth factor A
PFS: Progression-free survival
R2: Macroscopic residual tumour on resection
RECIST: Response Evaluation Criteria In Solid Tumours
RFA: Radio Frequency Ablation
SD: Stable disease
SDH: Succinate dehydrogenase
TG: Toxicity grade
TTP: Time to progression
VEGFR: Vascular endothelial growth factor receptor
WT: Wild type

## References

[1] Miettinen M, Lasota J (2006). Gastrointestinal stromal tumors: pathology and prognosis at different sites. Semin Diagn Pathol.

[2] Gatta G, van der Zwan JM, Casali PG, Siesling S, Dei Tos AP, Kunkler I (2011). Rare cancers are not so rare: the rare cancer burden in Europe. Eur J Cancer.

[3] Pappo AS, Janeway KA (2009). Pediatric gastrointestinal stromal tumors. Hematol Oncol Clin North Am.

[4] The ESMO/European Sarcoma Network Working Group (2014). Gastrointestinal stromal tumours: ESMO clinical practice guidelines for diagnosis, treatment and follow-up. Ann Oncol.

[5] Verweij J, Casali PG, Zalcberg J, LeCesne A, Reichardt P, Blay JY (2004). Progression-free survival in gastrointestinal stromal tumors with high-dose imatininb: randomised trial. Lancet.

[6] Zalcberg JR, Verveij J, Casali PG, LeCesne A, Reichardt P, Blay JY (2005). Outcome of patients with advanced gastro-intestinal stromal tumors crossing over to a daily imatinib dose of 800 mg after progression on 400 mg. Eur J Cancer.

[7] Blanke CD, Demetri GD, von Mehren M, Heinrich MC, Eisenberg B, Fletcher JA (2008). Long-term results from a randomized phase II trial of standard- versus higher-dose imatinib mesylate for patients with unresectable or metastatic gastrointestinal stromal tumors expressing KIT. J Clin Oncol.

[8] Blanke CD, Rankin C, Demetri GD, Ryan CW, von Mehren M, Benjamin RS (2008). Phase III randomized, intergroup trial assessing imatinib mesylate at two dose levels in patients with unresectable or metastatic gastrointestinal stromal tumors expressing the kit receptor tyrosine kinase: S0033. J Clin Oncol.

[9] Gastrointestinal Stromal Tumor Meta-Analysis Group (2010). (MetaGIST). Comparison of two doses of imatinib for the treatment of unresectable or metastatic gastrointestinal stromal tumors: a meta-analysis of 1,640 patients. J Clin Oncol.

[10] Le Cesne A, Ray-Coquard I, Bui BN, Adenis A, Rios M, Bertucci F (2010). Discontinuation of imatinib in patients with advanced gastrointestinal stromal tumors after 3 years of treatment: an open-label multicentre randomised phase 3 trial. Lancet Oncol..

[11] Demetri DG, van Oosterom AT, Garrett CR, Blackstein ME, Shah MH, Verweij J (2006). Efficacy and safety of sunitinib in patients with advanced gastrointestinal stromal tumor after failure of imatinib: a randomised controlled trial. Lancet.

[12] Reichardt P, Kang YK, Rutkowski P, Schuette J, Rosen LS, Seddon B (2015). Clinical outcomes of patients with advanced gastrointestinal stromal tumors: safety and efficacy in a worldwide treatment-use trial of sunitinib. Cancer.

[13] George S, Blay JY, Casali PG, Le Cesne A, Stephenson P, Deprimo SE (2009). Clinical evaluation of continuous daily dosing of sunitinib maleate in patients with advanced gastrointestinal tumor after imatinib failure. Eur J Cancer.

[14] Demetri GD, Reichardt P, Kang YK, Blay JY, Rutkowski P, Gelderblom H (2013). Efficacy and safety of regorafenib for advanced gastrointestinal stromal tumours after failure of imatinib and sunitinib (GRID): an international, multicentre, randomised, placebo-controlled, phase 3 trial. Lancet.

[15] Kang YK, Ryu MH, Yoo C, Ryoo B-Y, Kim HJ, Lee JJ (2013). Resumption of imatinib to control metastatic or unresectable gastrointestinal stromal tumours after failure of imatinib and sunitinib(RIGHT): a randomised, placebo-controlled, phase-3 trial. Lancet Oncol.

[16] Miettinen M, Lasota J, Sobin LH (2005). Gastrointestinal stromal tumors of the stomach in children and young adults: a clinicopathologic, immunohistochemical, and molecular genetic study of 44 cases with long-term follow-up and review of the literature. Am J Surg Pathol.

[17] Prakash S, Sarran L, Socci N, DeMatteo RP, Eisenstat J, Greco AM (2005). Gastrointestinal stromal tumors in children and young adults: a clinicopathologic, molecular, and genomic study of 15 cases and review of the literature. J Pediatr Hematol Oncol.

[18] Zhang L, Smyrk TC, Young WF Jr, Stratakis CA, Carney JA. Gastric stromal tumors in carney triad are different clinically, pathologically, and behaviorally from sporadic gastric gastrointestinal stromal tumors: findings in 104 cases. Am J Surg Pathol 2010;34:53–64. 10.1097/PAS.0b013e3181c20f4f PMC3652406 19935059

[19] Pasini B, McWhinney SR, Bei T, Matyakhina L, Stergiopoulos S, Muchow M (2008). Clinical and molecular genetics of patients with the carney-Stratakis syndrome and germline mutations of the genes coding for the succinate dehydrogenase subunits SDHB, SDHC, and SDHD. Eur J Hum Genet.

[20] Gaal J, Stratakis CA, Carney JA, Ball ER, Korpershoek E, Lodish MB (2011). SDHB immunohistochemistry: a useful tool in the diagnosis of carney-Stratakis and carney triad gastrointestinal stromal tumors. Mod Pathol.

[21] Boikos SA, Pappo AS, Killan JK, LaQuaglia MP, Weldon CB, George S (2016). Molecular subtypes of *KIT/PDGFRA* wild-type gastrointestinal tumors. A report from the National Institutes of Health gastrointestinal stromal tumor clinic. JAMA Oncol.

[22] Falor A, Arrington AK, Luu C, Schoelhammer HF, Ko M, Chow W, et al. Massive intra-abdominal imatinib-resistant gastrointestinal stromal tumor in a 21-year-old male. Case Rep Med. 2013; https://doi.org/10.1155/2013/373981. 10.1155/2013/373981 PMC3747627 23983708

[23] Janeway KA, Albritton KH, Van Den Abbeele AD, D’Amato GZ, Pedrazzoli P, Siena S (2009). Sunitinib treatment in pediatric patients with advanced GIST following failure of imatinib. Pediatr Blood Cancer.

[24] Gagnier J, Kienle G, Altman DG, Moher D, Sox H, Riley D (2013). The CARE guidelines: consensus-based clinical case report guideline development. J Clin Epidemiol.

[25] Wozniak A, Rutkowski P, Piskorz A, Ciwoniuk M, Osuch C, Bylina E (2012). Prognostic value of KIT/PDGFRA mutations in gastrointestinal stromal tumours (GIST): polish clinical GIST registry experience. Ann Oncol.

[26] Agaram NP, Laguaglia MP, Ustin B, Guo T, Wong GC, Socci ND (2008). Molecular characterization of pediatric gastrointestinal tumors. Clin Cancer Res.

[27] FDA USA. (United States of America food and drug administration). SUTENT® (sunitinib maleate) capsules. 2006. Available at: https://www.accessdata.fda.gov/drugsatfda_docs/label/2006/021968lbl.pdf. Accessed July 2017.

[28] Rutkowski P, Bylina E, Klimczak A, Świtaj T, Falkowski S, Kroc J (2012). The outcome and predictive factors of sunitinib therapy in advanced gastrointestinal stromal tumors (GIST) after imatinib failure - one institution study. BMC Cancer.

[29] de Wit D, van Erp NP, Khosravan R, Wiltshire R, Alfred R, Demetri GD (2014). Effect of gastrointestinal resection on sunitinib exposure in patients with GIST. BMC Cancer.

[30] Reichardt P, Demetri DG, Gelderblom H, Rutkowski P, Im SA, Gupta S (2016). Correlation of KIT and PDGFRA mutational status with clinical benefit in patients with gastrointestinal stromal tumor treated with sunitinib in a worldwide treatment-use trial. BMC Cancer.

[31] Pilotte AP (2015). Current management of patients with gastrointestinal stromal tumor receiving the multitargeted tyrosine kinase inhibitor sunitinib. Curr Med Res Opin.

